# Identification of Cholesterol in Plaques of Atherosclerotic Using Magnetic Resonance Spectroscopy and 1D U-Net Architecture

**DOI:** 10.3390/molecules31020352

**Published:** 2026-01-19

**Authors:** Angelika Myśliwiec, Dawid Leksa, Avijit Paul, Marvin Xavierselvan, Adrian Truszkiewicz, Dorota Bartusik-Aebisher, David Aebisher

**Affiliations:** 1 Department of Biochemistry and General Chemistry, Medical Faculty, Collegium Medicum, University of Rzeszów, 35-310 Rzeszów, Poland; amysliwiec@ur.edu.pl (A.M.); dbartusikaebisher@ur.edu.pl (D.B.-A.); 2 Rzeszów Center for Vascular and Endovascular Surgery, 35-310 Rzeszów, Poland; dleksa@gmail.com; 3 Department of Biomedical Engineering, Tufts University, Medford, MA 02155, USA; avijit.paul@tufts.edu (A.P.); marvin.xavierselvan@tufts.edu (M.X.); 4 Department of Photomedicine and Physical Chemistry, Medical Faculty, Collegium Medicum, University of Rzeszow, 35-310 Rzeszów, Poland; atruszkiewicz@ur.edu.pl

**Keywords:** cholesterol, MRS, atherosclerotic plaque, cholesterol synthesis, 1D U-Net

## Abstract

Cholesterol plays a fundamental role in the human body—it stabilizes cell membranes, modulates gene expression, and is a precursor to steroid hormones, vitamin D, and bile salts. Its correct level is crucial for homeostasis, while both excess and deficiency are associated with serious metabolic and health consequences. Excessive accumulation of cholesterol leads to the development of atherosclerosis, while its deficiency disrupts the transport of fat-soluble vitamins. Magnetic resonance spectroscopy (MRS) enables the detection of cholesterol esters and the differentiation between their liquid and crystalline phases, but the technical limitations of clinical MRI systems require the use of dedicated coils and sequence modifications. This study demonstrates the feasibility of using MRS to identify cholesterol-specific spectral signatures in atherosclerotic plaque through ex vivo analysis. Using a custom-designed experimental coil adapted for small-volume samples, we successfully detected characteristic cholesterol peaks from plaque material dissolved in chloroform, with spectral signatures corresponding to established NMR databases. To further enhance spectral quality, a deep-learning denoising framework based on a 1D U-Net architecture was implemented, enabling the recovery of low-intensity cholesterol peaks that would otherwise be obscured by noise. The trained U-Net was applied to experimental MRS data from atherosclerotic plaques, where it significantly outperformed traditional denoising methods (Gaussian, Savitzky–Golay, wavelet, median) across six quantitative metrics (SNR, PSNR, SSIM, RMSE, MAE, correlation), enhancing low-amplitude cholesteryl ester detection. This approach substantially improved signal clarity and the interpretability of cholesterol-related resonances, supporting more accurate downstream spectral assessment. The integration of MRS with NMR-based lipidomic analysis, which allows the identification of lipid signatures associated with plaque progression and destabilization, is becoming increasingly important. At the same time, the development of high-resolution techniques such as μOCT provides evidence for the presence of cholesterol crystals and their potential involvement in the destabilization of atherosclerotic lesions. In summary, nanotechnology-assisted MRI has the potential to become an advanced tool in the proof-of-concept of atherosclerosis, enabling not only the identification of cholesterol and its derivatives, but also the monitoring of treatment efficacy. However, further clinical studies are necessary to confirm the practical usefulness of these solutions and their prognostic value in assessing cardiovascular risk.

## 1. Introduction

Cholesterol is one of the key molecules in the human body—essential for the proper functioning of cells, but at the same time potentially harmful when present in excess or deficiency. Its role is most often associated with the stabilization of cell membranes, but in reality, it performs much broader and more complex biological functions. The foundation of its versatility is its unique chemical structure, which includes three essential elements: a hydrophilic part, a hydrophobic fragment, and a rigid steroid ring. This specific structure enables cholesterol to regulate many cellular processes, from controlling the fluidity and permeability of biological membranes to modulating the activity of transcription factors and gene expression [1].

**Figure 1:**
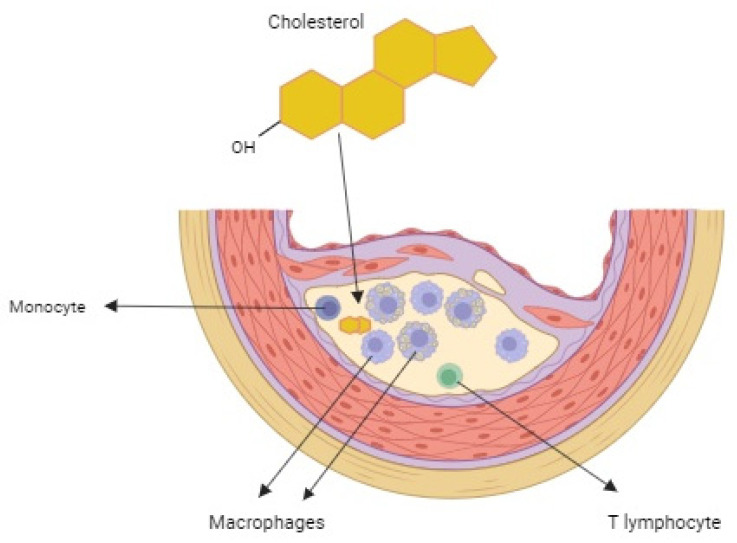
The formation of atherosclerosis with the participation of cholesterol.

Cholesterol not only acts as an independent regulatory molecule, but is also the basic precursor of all steroid hormones (such as estrogens, testosterone, and cortisol) and vitamin D derivatives (Figure 1). As a result, it plays a fundamental role in the processes of growth, maturation, and maintenance of homeostasis throughout life. A growing number of studies also point to its potential importance in cancer therapy as an element supporting the fight against cancer [2]. Interestingly, traces of pathological cholesterol accumulation have been observed in tissues dating back thousands of years—both in a 5000-year-old ice man and in ancient mummies from various cultures around the world [3]. On the other hand, cholesterol deficiency also has serious consequences. Too low a concentration of this compound in the blood disrupts the transport and distribution of fat-soluble vitamins, especially vitamins K and E, to key organs, which can result in serious metabolic and health disorders [4].

There are several classes of lipoproteins: chylomicrons, very low-density lipoproteins (VLDLs), intermediate-density lipoproteins (IDLs), low-density lipoproteins (LDLs), and high-density lipoproteins (HDLs). They differ in their cholesterol, triglyceride, and protein content, as well as in their biological function [5].

### 1.1. Cholesterol Synthesis: Molecular Pathway and Clinical Relevance

Cholesterol synthesis involves over 30 enzymatic steps converting acetyl-CoA into cholesterol, many of which are targets of lipid-lowering therapies. The pathway proceeds through the mevalonate pathway, regulated by HMG-CoA reductase, followed by the formation of farnesyl pyrophosphate, cyclization of squalene to lanosterol, and final modifications producing cholesterol. These stages are tightly controlled by the SREBP2–SCAP–INSIG regulatory system.

Cholesterol synthesis begins in the cytosol and endoplasmic reticulum with the condensation of acetyl-CoA. Thiolase catalyzes the formation of acetoacetyl-CoA, which is subsequently converted to HMG-CoA by HMG-CoA synthase. HMG-CoA reductase then catalyzes the rate-limiting step of the pathway [6].

HMG-CoA reductase (HMGCR), an endoplasmic reticulum-associated membrane enzyme, catalyzes the reduction of HMG-CoA to mevalonate (MVA) using NADPH. This reaction represents the rate-limiting and principal regulatory step of cholesterol biosynthesis and is the primary therapeutic target of statins, which act as competitive inhibitors and reduce hepatic cholesterol synthesis [7].

Mevalonate subsequently undergoes phosphorylation and decarboxylation to form activated isoprenoid units. Mevalonate is converted to mevalonate-5-phosphate by mevalonate kinase, then to mevalonate-5-pyrophosphate by phosphomevalonate kinase, and finally decarboxylated to isopentenyl pyrophosphate (IPP) by mevalonate pyrophosphate decarboxylase. IPP is isomerized to dimethylallyl pyrophosphate (DMAPP), and together these isoprenoids serve as essential building blocks for squalene and cholesterol synthesis [7,8].

The mevalonate pathway is a central regulatory node supplying isoprenoid intermediates for sterol and non-sterol compounds, including ubiquinone, dolichols, and protein prenylation, and is tightly controlled by intracellular cholesterol levels and lipid-lowering therapies [9]. Following the formation of activated isoprenoid units—IPP and DMAPP—the pathway enters the prenyl condensation phase in the cytosol. DMAPP condenses with IPP to form geranyl pyrophosphate (GPP), which subsequently reacts with another IPP molecule to generate farnesyl pyrophosphate (FPP). FPP represents a major metabolic branch point, serving as a precursor for cholesterol as well as numerous non-sterol products [9,10].

FPP contributes to the biosynthesis of ubiquinone, dolichols, heme A, and provides prenyl groups for post-translational modification of signaling proteins such as RAS and Rho family members, thereby dividing isoprenoid flux between sterol and non-sterol pathways [11]. In cholesterol synthesis, two FPP molecules condense to form squalene in a reaction catalyzed by squalene synthase (FDFT1), an endoplasmic reticulum enzyme that directs isoprenoids toward sterol production [12]. Because squalene synthase acts downstream of the mevalonate pathway, its inhibition has been explored as a cholesterol-lowering strategy that preserves non-sterol isoprenoid synthesis, although clinical development of some inhibitors has been limited by adverse effects [13].

Squalene then undergoes cyclization reactions in the endoplasmic reticulum. First, squalene epoxidase (SQLE) converts squalene to 2,3-epoxysqualene in a cholesterol-regulated, NADPH-dependent reaction representing a second major control point of the pathway [7]. Subsequently, lanosterol synthase (LSS) catalyzes the cyclization of 2,3-epoxysqualene to lanosterol, forming the characteristic tetracyclic sterol skeleton conserved across sterol-producing organisms [14]. Clinically, SQLE inhibitors and mutations in LSS have been linked to lipid disorders, developmental abnormalities, and skin diseases [15,16,17,18].

Following lanosterol formation, cholesterol biosynthesis proceeds through multiple demethylation, reduction, and isomerization reactions catalyzed mainly by microsomal oxidoreductases and cytochrome P450 enzymes. Two alternative routes operate at this stage: the Bloch pathway and the Kandutsch–Russell pathway, which differ in the timing of Δ24 double-bond reduction in the sterol side chain [19,20,21]. Key enzymes include CYP51A1 for C14 demethylation, SC4MOL, NSDHL, and HSD17B7 for C4 demethylation, and several reductases and isomerases that finalize cholesterol formation [21,22].

The Bloch pathway, prominent in the liver, yields desmosterol as the final intermediate, whereas the Kandutsch–Russell pathway, important in the brain and skin, produces 7-dehydrocholesterol, whose accumulation due to DHCR7 deficiency causes Smith–Lemli–Opitz syndrome [19,20,21]. Importantly, cells can dynamically switch between these pathways depending on tissue type and metabolic conditions [23]. Additionally, a minor shunt of the mevalonate pathway generates 24(S),25-epoxycholesterol via alternative SQLE and LSS activity, producing a bioactive oxysterol involved in sterol regulation [24].

24(S),25-epoxycholesterol functions as an endogenous ligand for liver X receptors (LXRs), activating genes involved in cholesterol efflux, including ABCA1 and ABCG1. In this way, it links cholesterol biosynthesis with its cellular removal and acts as an important regulatory signaling molecule rather than a mere byproduct of sterol synthesis [25]. Sterol intermediates, including oxysterols, also regulate the stability of key enzymes such as HMG-CoA reductase (HMGCR) by promoting its ubiquitination and proteasomal degradation, thereby providing feedback control of cholesterol synthesis [26]. Disruption of these regulatory branches, including impaired 24(S),25-epoxycholesterol production, has been associated with altered LXR signaling and the development of metabolic and neurodegenerative diseases [27].

Cholesterol biosynthesis is regulated at multiple levels, with a central role played by the SREBP2–SCAP–INSIG system (Table 1). Under low sterol conditions, SREBP2 is activated and induces transcription of genes involved in cholesterol synthesis and uptake, including HMGCR, SQLE, and LDLR. At high cholesterol levels, SREBP2 activation is suppressed through retention in the endoplasmic reticulum by INSIG proteins [28,29]. Epigenetic regulation further modulates this pathway; for example, SIRT6 and FoxO_3_ repress Srebp2 transcription via histone deacetylation, limiting hepatic sterol synthesis and protecting against hypercholesterolemia [30].

Post-translational regulation is also critical, as HMGCR and SQLE undergo sterol-dependent degradation through the ER-associated degradation (ERAD) pathway, a process enhanced by specific sterol intermediates [31]. Additionally, cellular energy status influences cholesterol synthesis via AMPK-mediated phosphorylation and inhibition of HMGCR during energy deficiency [7]. Finally, microRNAs provide an additional post-transcriptional regulatory layer by modulating the expression of sterol biosynthetic enzymes, cholesterol transporters, and key transcriptional regulators, further fine-tuning cholesterol homeostasis [32].

### 1.2. Cholesterol Biosynthesis as an Important Clinical and Pharmacological Target

Cholesterol biosynthesis is a major clinical and pharmacological target due to its central role in cardiovascular disease and cancer. Excess plasma cholesterol, particularly LDL, promotes atherosclerosis and cardiovascular events, making pharmacological control essential. Statins, the most widely used cholesterol-lowering drugs, competitively inhibit HMG-CoA reductase (HMGCR), the rate-limiting enzyme of the mevalonate pathway, thereby reducing hepatic cholesterol synthesis and plasma LDL levels [33,34]. In addition, statins upregulate hepatic LDL receptors, enhancing LDL clearance from circulation and further lowering cholesterol concentrations [35].

Beyond lipid-lowering, statins exert pleiotropic effects, including anti-inflammatory activity, improved endothelial function via increased nitric oxide bioavailability, reduced oxidative stress, and stabilization of atherosclerotic plaques. These effects translate into a significant reduction in cardiovascular events and are well documented in clinical trials and guidelines [36,37]. Moreover, by limiting the production of isoprenoid intermediates such as farnesyl pyrophosphate (FPP) and geranylgeranyl pyrophosphate (GGPP), statins can impair prenylation of RAS and Rho proteins, thereby influencing cell proliferation and migration and highlighting their potential role in oncology [37,38].

An alternative therapeutic strategy targets squalene synthase (FDFT1), which catalyzes the conversion of FPP to squalene. Inhibitors of this enzyme, such as lapaquistat, act downstream of HMGCR and reduce cholesterol synthesis while preserving non-sterol isoprenoid production, potentially offering a different safety profile, particularly in statin-intolerant patients [39]. Because sterol intermediates like FPP and GGPP are essential for protein prenylation and oncogenic signaling, modulation of the mevalonate pathway—either at early or late stages—may limit cancer cell proliferation and invasiveness, making cholesterol biosynthesis an emerging therapeutic target in oncology [40,41].

Understanding the complexity and regulation of cholesterol biosynthesis underscores the importance of accurate, non-invasive detection methods.

### 1.3. The Importance of Cholesterol in the Development of Atherosclerosis

Cholesterol transported by LDL plays a central role in the initiation and progression of atherosclerosis. Excess circulating LDL penetrates the vascular endothelium and accumulates in the arterial intima, where it becomes trapped in the extracellular matrix and forms early lipid deposits. Macrophages take up native and modified LDL via scavenger receptors, leading to foam cell formation and the development of fatty streaks, the earliest atherosclerotic lesions [42,43].

Within the vessel wall, LDL undergoes oxidative and other modifications, generating oxLDL, which strongly promotes inflammation, monocyte recruitment, and smooth muscle cell (SMC) proliferation. OxLDL is internalized by macrophages and SMCs mainly through receptors such as CD36 and LOX-1, enhancing foam cell formation and contributing to plaque growth and instability [44,45,46,47]. SMCs can migrate into the intima and adopt a foam cell-like phenotype under lipid overload, further accelerating lesion progression [48].

Atherosclerosis is therefore a chronic inflammatory process driven by cholesterol accumulation. Excess intracellular cholesterol leads to the formation of insoluble cholesterol crystals, which induce cellular stress and amplify inflammation within plaques [49,50]. Cholesterol crystals activate the NLRP3 inflammasome in macrophages through lysosomal damage, cathepsin release, and endolysosomal cholesterol accumulation, while endoplasmic reticulum stress and complement activation further contribute to inflammasome signaling [51,52,53].

### 1.4. Nanotechnology in Detection of Cholesterol

Cholesterol is one of the most important and abundant steroids in the human body. It is an essential component of cell membranes and participates in regulating their fluidity and stability. Particularly high concentrations are observed in the brain and muscle tissue. Cholesterol also plays a key role as a precursor in the biosynthesis of many steroid hormones, such as adrenal cortex hormones (e.g., cortisol, aldosterone) and sex hormones (estrogens, androgens, progesterone). Additionally, it participates in the formation of bile acids and vitamin D, and also influences the integrity and functionality of blood vessels [54,55]. Despite these important functions, excessive cholesterol in the blood is a serious risk factor for the development of cardiovascular disease. Abundant epidemiological and clinical data indicate that elevated total cholesterol levels (the accepted reference range is approximately 120–260 mg/dL) significantly correlate with the occurrence of coronary heart disease, atherosclerosis, and peripheral vascular disease. Therefore, cholesterol testing is a key element of screening and diagnostics in cardiology, as well as in preventive healthcare [56,57].

In recent decades, the intensive development of nanotechnology has led to the creation of a wide range of advanced functional nanomaterials with diverse morphologies and properties. Zero-dimensional (0D) structures have been developed, such as nanoparticles and quantum dots; one-dimensional (1D), including nanowires, nanorods, and nanotubes; two-dimensional (2D), including transition metal oxides and dichalcogenides, as well as graphene-based materials; and three-dimensional (3D), which include hierarchical nanostructures in the form of flowers, cubes, or spheres. Various synthesis techniques are used to produce them, such as chemical vapor deposition (CVD), laser ablation, electrospinning, electrodeposition, hydrothermal methods, vapor phase transport, sol–gel processes, or thermal evaporation [58,59,60,61].

Nanostructures obtained this way are characterized by unique properties resulting from precise control of their morphology, large specific surface area, and significantly improved electrocatalytic parameters. This makes them superior to traditional materials in terms of activity, stability, and integration potential in functional systems. Over the past decade, their applications have been rapidly expanding—from energy storage and conversion systems (lithium-ion batteries, supercapacitors, fuel cells), through photonics and optoelectronics, to the fabrication of modern sensors, including non-enzymatic sensors for the detection of biomolecules and environmental pollutants.

**Figure 2:**
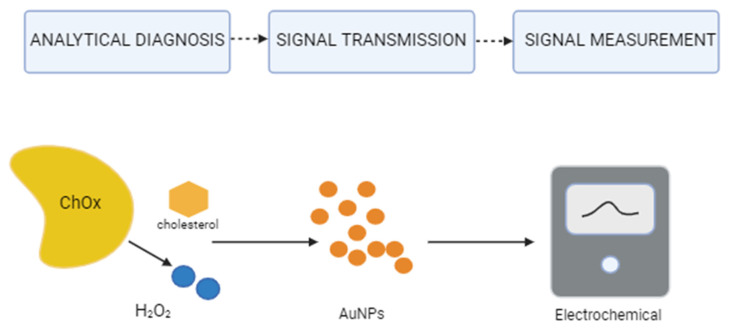
Detecting cholesterol using nanotechnology.

Among nanomaterials, graphene is particularly noteworthy—a two-dimensional material composed of sp^2^-hybridized carbon atoms arranged in a honeycomb structure (Figure 2). It is characterized by excellent biocompatibility, exceptionally high electrical conductivity, a large specific surface area, and a zero energy gap, making it an excellent platform for creating highly effective biosensors. Its derivative, reduced graphene oxide (rGO), offers even greater application potential. Due to the presence of numerous functional groups, such as hydroxyl and carboxyl groups, rGO enables a variety of chemical interactions and facilitates enzyme immobilization, significantly increasing the stability and sensitivity of electrochemical biosensors for cholesterol determination [62,63,64]. Zhu et al.’s study developed a new biosensor for cholesterol detection, utilizing gold nanoparticles (Au NPs) and the rGO-PAMAM-Fc hybrid nanomaterial. Cholesterol oxidase (ChOx) and cholesterol esterase (ChEt) were immobilized on the surface of the modified electrode, creating the ChOx&ChEt/Au NPs/rGO-PAMAM-Fc biosensor. The resulting device was characterized by high stability, selectivity, and repeatability, and under optimal conditions, it showed a linear response in the range of 0.0004–15.36 mM (R^2^ = 0.9986) and a very low detection limit of 2 nM. Importantly, the biosensor was successfully used to analyze cholesterol in human serum samples, confirming its potential in clinical diagnostics [65].

Accurate cholesterol measurement is essential for the diagnosis and monitoring of numerous diseases, including cardiovascular and metabolic disorders. Traditional electrochemical sensors often suffer from limited sensitivity and selectivity, prompting the development of advanced biosensors with improved analytical performance.

Kumar et al. developed a localized surface plasmon resonance (LSPR)-based cholesterol biosensor using a conical optical fiber functionalized with gold nanoparticles (AuNPs) and cholesterol oxidase. Comprehensive physicochemical characterization confirmed the uniformity and stability of the nanostructures, and the sensor demonstrated high sensitivity across a clinically relevant concentration range (0–10 mM) with a detection limit of 53.1 nM [66]. Zhang et al. reported an electrochemical biosensor incorporating Au and Pt nanoparticles and a PAMAM–ZIF-67 hybrid material, with immobilized cholesterol oxidase and esterase. The device exhibited a wide linear range (0.00015–10.24 mM), a low detection limit of 3 nM, and high accuracy in human serum samples, highlighting its clinical potential [67]. Elhag et al. introduced Co_3_O_4_ nanostructures with a cotton-like morphology, offering a large active surface area, high stability, biocompatibility, and rapid response, which together enabled sensitive and reliable cholesterol detection [68]. Khaliq et al. demonstrated a non-enzymatic cholesterol biosensor based on TiO_2_ nanotubes decorated with CuO_2_ nanoparticles. Compared to unmodified nanotubes, the hybrid TiO_2_/CuO_2_ sensor showed enhanced electrochemical performance, including higher sensitivity, faster response, and improved selectivity, as well as stability under near-physiological conditions, supporting its applicability in clinical diagnostics [69]. In summary, the use of nanomaterials in the construction of cholesterol sensors opens up new possibilities for the rapid, sensitive, and reliable detection of this important biomarker. A variety of structures—from nanotubes and nanoparticles to complex hybrid systems—allows for precise tuning of surface properties, increased conductivity, and improved chemical and biological stability of the sensors. Thanks to unique features such as large active surface area, biocompatibility, non-toxicity, and the ability to catalytically accelerate reactions, nanomaterials significantly outperform traditional electrode materials. Furthermore, their integration with non-enzymatic methods eliminates limitations related to enzyme sensitivity to environmental conditions, making these solutions particularly attractive for practical applications in clinical diagnostics and health monitoring.

### 1.5. Scope of the Present Study

While nanotechnology-based approaches show considerable promise for enhancing MRI contrast and specificity in cholesterol detection, the fundamental challenge remains the reliable identification of cholesterol-specific spectral signatures using magnetic resonance spectroscopy (MRS). Current clinical MRI systems, despite their widespread availability, face significant limitations in detecting and quantifying cholesterol within atherosclerotic plaques. These limitations stem primarily from insufficient spatial and spectral resolution at standard clinical field strengths (1.5–3 T), as well as the lack of specialized receiving coils optimized for small-volume lipid detection. Our experimental design employs a custom-developed receiving coil specifically adapted for small-volume specimens, coupled with modified spectroscopic sequences. By dissolving atherosclerotic plaque material in deuterated chloroform, a standard solvent in high-resolution NMR spectroscopy, we aimed to establish whether the spectral fingerprint of plaque-derived cholesterol could be unambiguously identified and matched to established NMR databases. This ex vivo validation represents a necessary first step before attempting more complex in vivo translation. This study, therefore, serves as a proof-of-concept investigation, establishing the technical feasibility of MRS-based cholesterol detection in atherosclerotic tissue under optimized ex vivo conditions. While clinical translation will require substantial additional development, including in vivo validation, motion compensation strategies, and potentially the integration of contrast-enhancing nanoparticles, the fundamental spectroscopic characterization presented here provides an essential foundation for such future work.

### 1.6. Comparison of MRS and 1D U-Net Deep Learning Model with Traditional Methods

Cholesterol, especially in the form of cholesteryl esters, plays a key role in the development of atherosclerotic plaques, and its accurate detection in tissue is essential for understanding the mechanisms of atherosclerosis progression and evaluating the effectiveness of lipid-lowering therapies. Traditional cholesterol analysis techniques, such as high-resolution NMR or lipid extraction according to the Bligh-Dyer or Folch protocols, enable precise quantitative determination of lipids in homogenized tissue samples. However, these methods are invasive, require time-consuming sample processing, and the homogenization and multi-step extraction process can lead to lipid loss or modification of their chemical structure [70,71].

Proton nuclear magnetic resonance spectroscopy (^1^H-MRS) is an attractive alternative, enabling non-invasive detection of cholesterol and cholesteryl esters without the need to destroy tissue. This technique preserves the spatial context of lipids in atherosclerotic plaques, which is difficult to achieve with classical chemical extractions. However, the interpretation of MRS spectra is sometimes hampered by a low signal-to-noise ratio, broad and overlapping lipid resonances, and limited sensitivity in samples with low cholesterol content [70,72].

Combining MRS with deep learning algorithms, such as the 1D U-Net model, significantly improves cholesterol detection capabilities in tissues. Neural networks enable automatic spectrum deconvolution, signal calibration, and peak segmentation, improving the detectability of subtle resonances in spectra with low signal-to-noise ratios [72]. This approach eliminates subjective operator errors, reduces the time required to analyze large data sets, and allows for the repeatable identification of characteristic cholesterol proton signals while preserving information about the local distribution of lipids in plaques.

Thanks to these properties, MRS supported by the 1D U-Net model offers unique advantages over classical methods: it enables the detection of cholesterol at its physiological site of occurrence, increases the sensitivity and accuracy of the analysis, allows for the automation of spectrum processing, and enables the study of lipid heterogeneity within the tissue. This approach is particularly important in studies on the progression of atherosclerosis, where subtle changes in cholesterol content can be clinically significant but may be overlooked in chemical extractions or distorted by prolonged homogenization and multi-day extraction. Recently, other deep learning-based studies also incorporated various deconvolution methods into MRS, such as [73,74,75,76,77].

In summary, the integration of MRS with the 1D U-Net algorithm provides a modern and non-invasive tool for analyzing cholesterol in atherosclerotic plaques, combining the advantages of traditional quantitative methods with the ability to preserve spatial context, automate analysis, and improve detection sensitivity and precision. This approach can be used in both basic research and clinical applications, contributing to a better understanding of the role of lipids in the progression of atherosclerosis and the evaluation of the effectiveness of lipid-lowering therapies.

## 2. Results

### 2.1. Spectral Acquisition from Pure Cholesterol Standard

**Figure 3:**
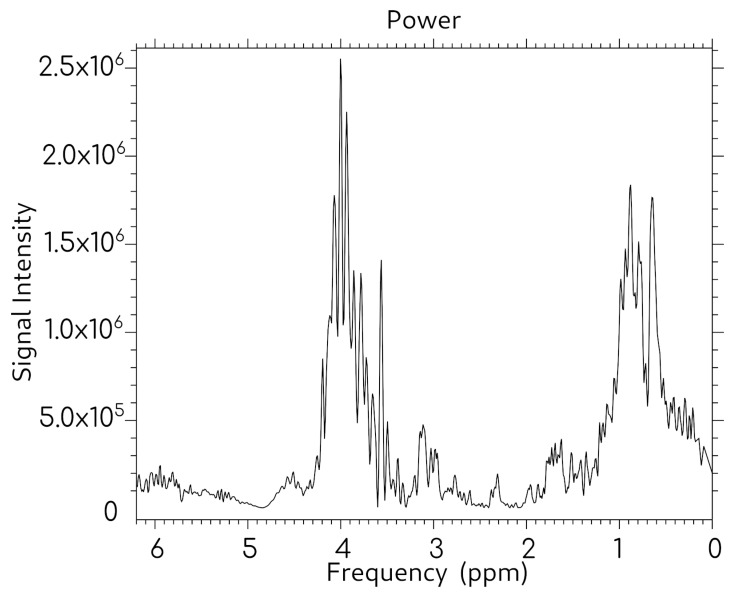
Spectrum of a solution of 0.2 mg of cholesterol in 2 mL of chloroform. Sequence parameters: TR = 1500 ms, TE = 35 ms.

To establish baseline spectral characteristics and optimize acquisition parameters, an initial validation experiments were performed using pure cholesterol dissolved in chloroform. Figure 3 presents the MRS spectrum obtained from 0.2 mg of cholesterol dissolved in 2 mL of chloroform, acquired using a TR of 1500 ms and TE of 35 ms. The spectrum clearly demonstrates the presence of characteristic cholesterol resonances, confirming the capability of the modified receiving coil system and acquisition sequences to detect cholesterol-specific signals in this solvent system.

### 2.2. Time-Dependent Spectral Evolution of Atherosclerotic Plaque

Atherosclerotic plaque tissue was fragmented and immersed in chloroform, and MRS spectra were acquired at two time points to assess dissolution kinetics and spectral development. Spectra were obtained using TR = 1200 ms and TE = 28 ms.

**Figure 4:**
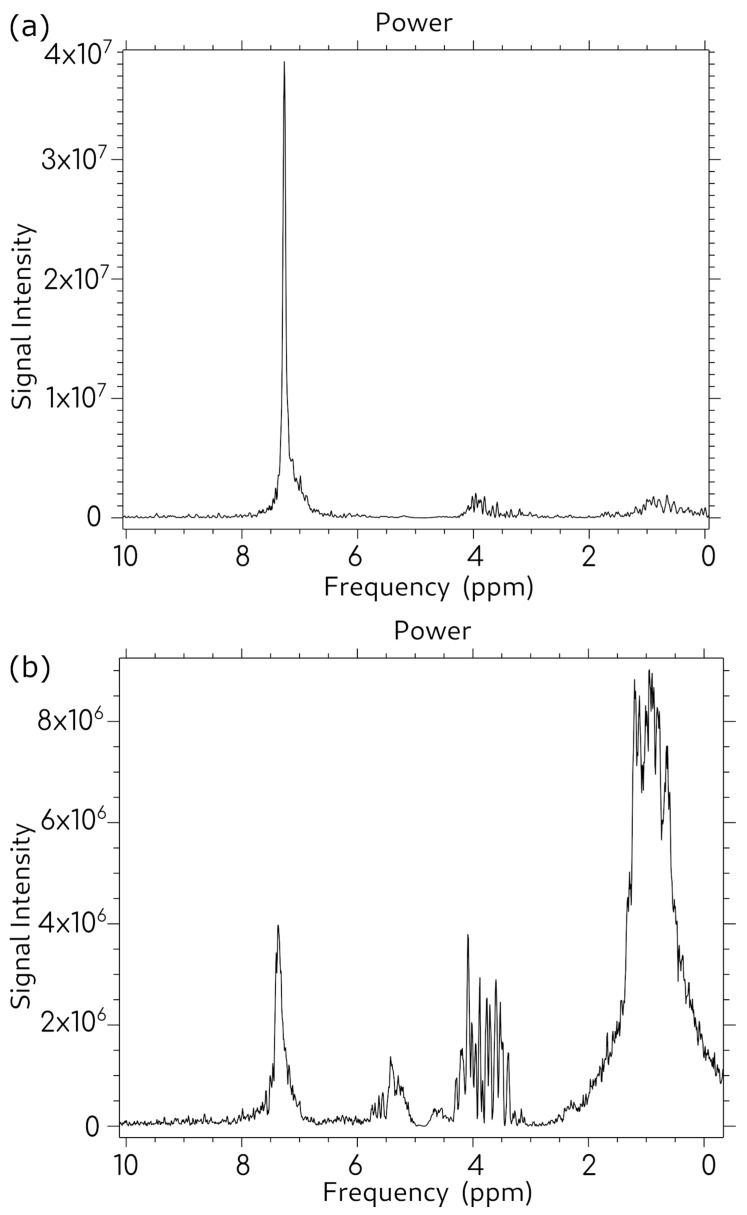
Spectrum of the same tube containing fragmented atherosclerotic plaque immersed in chloroform. (**a**) State 1 h after immersion, (**b**) state 7 days after immersion. Sequence parameters TR = 1200 ms, TE = 28 ms.

Figure 4a shows the spectrum acquired one hour after plaque immersion in chloroform. At this early time point, the spectrum is dominated by a high-intensity chloroform peak at 7.25 ppm, with a minimal detectable cholesterol signal. This observation indicates that the plaque material had not yet undergone significant dissolution, and cholesterol extraction into the solvent remained incomplete.

Figure 4b presents the spectrum of the same sample after seven days of dissolution. A dramatic transformation in spectral characteristics is evident. Multiple well-resolved cholesterol peaks emerged with intensities that significantly exceeded the chloroform solvent signal. This marked enhancement demonstrates that extended dissolution time allows for more complete cholesterol extraction from the atherosclerotic plaque matrix, resulting in substantially improved signal detection.

At the same time, it should be emphasized that the observed extraction kinetics were the result of several days of tissue contact with chloroform, without the use of mechanical homogenization. In standard lipid isolation methods, such as the Bligh-Dyer or Folch protocols, the tissue is pre-homogenized, e.g., using a Dounce homogenizer, which allows for rapid and efficient release of lipids into the solvent in a much shorter time. This approach not only shortens the extraction time but also ensures more controlled and repeatable lipid isolation, reducing the risk of incomplete extraction due to limited diffusion in the tissue matrix.

### 2.3. Cholesterol Peak Identification and Assignment

To validate the cholesterol-specific nature of the observed resonances, spectral peak positions were compared against reference NMR data from the Human Metabolome Database (HMDB entry 2491). Table S1 (Supporting Information) presents the complete reference dataset, including resonance frequencies (Hz), chemical shifts (ppm), relative intensities, and assigned signal numbers corresponding to specific structural features of the cholesterol molecule.

**Figure 5:**
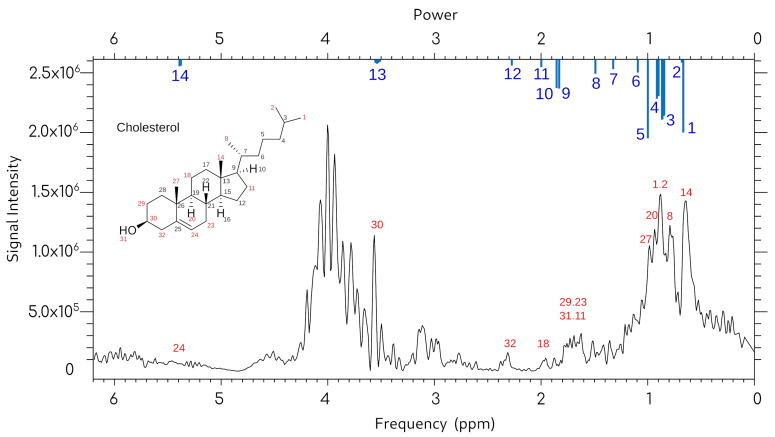
Spectrum of the tested sample, atherosclerotic plaque dissolved in chloroform, with identified peaks marked. Signals marked in blue correspond to signals from the online database. Initial spectrum in the 1.5 T (63.885 MHz) system-type: OPTIMA 360 MR, manufactured by GEMS. The spectrum is of the “Power” type. This is an unmodified spectrum, and there was no modification of the baseline.

Figure 5 presents the experimental spectrum with identified peaks annotated in blue, indicating signals that correspond to characteristic cholesterol resonances documented in the reference database. Of the fourteen characteristic cholesterol peaks present in the reference spectrum, we successfully identified and matched key resonances in the plaque-derived sample, providing robust confirmation of cholesterol presence in the atherosclerotic tissue.

The identified peaks include the characteristic olefinic proton signal at approximately 5.35 ppm (corresponding to the C5-C6 double bond in the steroid ring system), as well as multiple aliphatic resonances in the 0.7–2.3 ppm region corresponding to methyl and methylene protons throughout the cholesterol structure. The spectral pattern and relative peak intensities closely matched those of the reference standard, confirming successful detection and characterization of plaque-derived cholesterol using the modified MRS system.

The most intense signal (peak 5, intensity = 1000) appears at 1.007 ppm and arises from overlapping methyl and methylene groups in the steroid core. Diagnostically valuable isolated resonances include peak 14 at 5.35 ppm (olefinic proton at the C5-C6 double bond), which is highly characteristic of cholesterol, and peak 13 at 3.5 ppm (C3 hydroxyl-bearing methine proton). Mid-field signals (peaks 9–12, 1.8–2.3 ppm) represent protons adjacent to functional groups or ring junctions, while upfield signals (peaks 1–4, below 1.0 ppm) correspond to angular methyl groups (C18, C19) and side-chain methyls.

**Figure 6:**
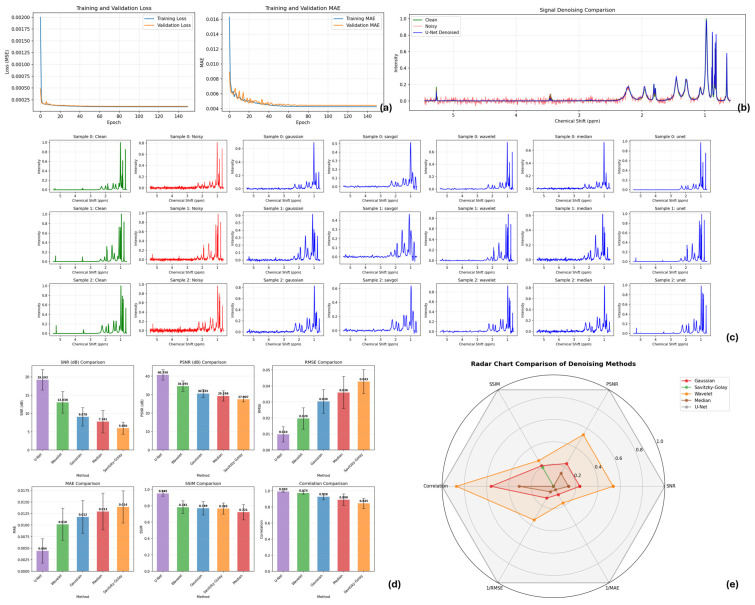
Comprehensive evaluation of the 1D U-Net denoiser on experimental MRS plaque spectra: (**a**) Training and validation curves for mean squared error (MSE, left axis) and mean absolute error (MAE, right axis) over 80 epochs, showing stable convergence without overfitting. (**b**) Representative raw experimental MRS spectrum from an atherosclerotic plaque specimen obscured by noise and baseline drift. The corresponding clean signal is denoted with green color and U-Net denoised spectra with blue. (**c**) Denoising comparison of a selected spectral region (0–6 ppm). From left to right: clean signal; noisy input; Gaussian smoothing (σ=1.5); Savitzky–Golay filter (window = 21, order = 3); wavelet denoising (Daubechies 4, soft thresholding); median filter (kernel = 5); and U-Net output. The U-Net best preserves peak shapes while suppressing noise. (**d**) Bar plot comparing the average performance of each denoising method across six quantitative metrics: SNR, PSNR, RMSE, MAE, SSIM, and Pearson correlation coefficient (mean ± SD). The U-Net achieves the highest scores for SNR, PSNR, SSIM, and correlation, and the lowest RMSE and MAE. (**e**) Radar chart visualizing the normalized performance of each method across the same six metrics (higher values indicate better performance). The U-Net (grey) encloses the largest area, demonstrating its overall superiority over traditional approaches.

Cross-referencing the experimental plaque spectrum (Figure 6) with these reference assignments enabled definitive identification of cholesterol-derived signals. Peaks marked in blue in Figure 5 represent resonances unambiguously matched to reference data in Table S1 (Supporting Information), confirming cholesterol presence in the atherosclerotic plaque sample.

Figure 5 presents the experimental plaque spectrum with identified cholesterol peaks marked in blue, corresponding to peak numbers in Table S1 (Supporting Information). Multiple diagnostic cholesterol resonances were successfully identified, including peak 5 at approximately 1.0 ppm (intense aliphatic envelope), peaks 6–8 (1.1–1.5 ppm region), peaks 10–12 (1.85–2.3 ppm), peak 14 at 5.35 ppm (olefinic proton at C5-C6 double bond), and peak 13 at 3.5 ppm (hydroxyl-bearing methine). Peak 14 provides particularly reliable identification due to its location in an uncrowded spectral region. Not all fourteen reference peaks could be identified in the experimental spectrum due to lower spectral resolution at 1.5 T compared to the 400 MHz reference system and potential overlap with other plaque components. The identified resonances confirm successful cholesterol detection in atherosclerotic plaque material.

### 2.4. Denoising MRS Spectra for Clear Peak Identification

The proposed 1D U-Net denoiser demonstrated robust performance in both synthetic and experimental MRS data, significantly improving spectral quality and enabling clearer identification of low-amplitude lipid resonances in atherosclerotic plaque spectra.

Training convergence and model stability (Figure 6a): The model achieved rapid and stable convergence during training, as shown in Figure 6a. Both training and validation losses—mean squared error (MSE) and mean absolute error (MAE) decreased steadily over 150 epochs without signs of overfitting. The close alignment between training and validation curves indicates strong generalization to unseen spectra. Final validation MSE reached 2.1580 × 10^−4^ and MAE reached 0.0075, confirming the model’s capacity to learn noise-to-clean mappings effectively.

Denoising performance on experimental plaque spectra (Figure 6b): Figure 6b illustrates a representative raw experimental MRS spectrum acquired from an atherosclerotic plaque specimen. The spectrum exhibits characteristic broad lipid resonances superimposed on a noisy, drifting baseline. After U-Net processing (blue trace), the baseline stabilizes and low-amplitude cholesteryl ester signals near 2.8–6 ppm become discernible, closely matching the synthetic clean reference (green trace). Visual inspection confirms that the denoiser suppresses high-frequency noise while preserving the intrinsic lineshapes of lipid peaks.

Comparative analysis with traditional denoising methods (Figure 6c): We compared the U-Net against four conventional denoising approaches: Gaussian smoothing, Savitzky–Golay filtering, wavelet denoising, and median filtering. Figure 6c presents a detailed view of the 0–6 ppm region processed by each method. While all traditional techniques reduced noise to varying degrees, they often introduced undesirable artifacts: Gaussian smoothing overly broadened peaks, Savitzky–Golay filtering attenuated subtle metabolic features, wavelet denoising created pseudo-peaks near sharp transitions, and median filtering distorted peak symmetry. In contrast, the U-Net output retained the morphological fidelity of the clean reference while achieving superior noise suppression.

Quantitative evaluation across multiple metrics (Figure 6d,e): A comprehensive quantitative assessment was performed using six established metrics: signal-to-noise ratio (SNR), peak signal-to-noise ratio (PSNR), structural similarity index (SSIM), root mean square error (RMSE), MAE, and Pearson correlation coefficient. Results averaged over all experimental plaque spectra are summarized in Figure 6d (bar plot) and Figure 6e (radar chart). The radar chart visualizes the normalized performance profile of each method across all six metrics, with the U-Net enclosing the largest area, indicating balanced superiority in every dimension. The U-Net consistently outperformed all baseline methods, achieving the highest SNR, PSNR, SSIM, and correlation coefficient, along with the lowest RMSE and MAE. Paired *t*-tests confirmed that these improvements were statistically significant (*p* < 0.01) for all metrics versus each traditional method.

Recovery of low amplitude metabolic signatures: Of particular clinical relevance is the denoiser’s ability to recover faint cholesteryl ester resonances that are otherwise buried in the noise floor. In multiple plaque spectra, peaks in the 2.6–5.8 ppm range—indicative of esterified cholesterol-were visually obscured in raw data but clearly emerged after U-Net processing.

The denoiser maintained consistent performance across spectra with varying degrees of baseline drift, line broadening, and noise profiles, reflecting the diversity inherent in clinical MRS acquisitions. No manual parameter tuning was required for individual spectra, underscoring the model’s applicability as a fully automated preprocessing tool. The close alignment between training and validation loss curves indicates that the network generalized well to unseen spectra and did not exhibit overfitting. Furthermore, the model improved spectral smoothness without distorting peak positions or shapes, a critical requirement for downstream metabolite quantification (Table 2).

The denoised spectra showed marked improvement in the visibility of low-amplitude resonances, particularly in crowded spectral regions where overlapping peaks are typically obscured by noise. Baseline distortions, which often interfere with accurate peak integration, were effectively suppressed. Importantly, the network preserved subtle metabolic signatures and avoided excessive smoothing, suggesting that the model successfully learned metabolite-specific structural patterns rather than applying generic filtering.

## 3. Discussion

### 3.1. Interpretation of Experimental Findings

This study successfully identified 14 cholesterol-specific spectral peaks in ex vivo atherosclerotic plaque samples using clinical MRI equipment with a custom-designed receiving coil. As shown in Figure 6 and Table S1 (Supporting Information), the spectral signatures obtained from dissolved plaque material demonstrated excellent correspondence with reference cholesterol spectra from the Human Metabolome Database (400 MHz NMR). The identification of these characteristic peaks—ranging from 0.678 ppm to 5.356 ppm—confirms the feasibility of cholesterol detection using magnetic resonance spectroscopy in atherosclerotic tissue, even when using a standard clinical 1.5 T field strength MRI system.

A critical technical achievement of this work was the adaptation of the receiving system to enable spectroscopic analysis of small-volume samples. Standard clinical MRI coils designed for human organ imaging are unsuitable for analyzing 2 mL samples in Eppendorf or NMR tubes. The custom experimental coil developed for this study (previously described by our research team [78] was specifically optimized for small objects, with modified voxel dimensions of 8 × 8 × 8 mm (512 mm^3^) compared to the typical 20 × 20 × 20 mm (8000 mm^3^) used in brain spectroscopy. While this smaller voxel results in weaker signal intensity, the specialized coil geometry provided sufficient signal-to-noise ratio to detect cholesterol peaks in the dissolved plaque samples. This technical modification demonstrates that clinical MRI systems can be adapted for ex vivo spectroscopic studies of atherosclerotic material, bridging laboratory analysis with clinical imaging capabilities.

The time-dependent dissolution results (Figure 5) revealed important insights into sample preparation methodology. Initial measurements taken 1 h after immersion of fragmented atherosclerotic plaque in chloroform showed predominantly solvent signal (7.25 ppm) with minimal cholesterol peaks, indicating insufficient dissolution time. After 7 days, however, the cholesterol signals dramatically increased and exceeded the solvent signal, demonstrating complete extraction of cholesterol from the tissue matrix. This finding has significant implications for standardizing sample preparation protocols in MRS-based plaque analysis. The extended dissolution time required suggests that cholesterol in atherosclerotic tissue exists in a complex, matrix-bound state rather than as freely accessible lipid pools. Future studies should account for this dissolution kinetics when developing protocols for ex vivo spectroscopic analysis, and investigation of accelerated extraction methods may be warranted for clinical laboratory applications.

Using 0.2 mg cholesterol dissolved in 2 mL chloroform, we obtained a clear spectral pattern that enabled direct comparison with the plaque-derived signals. The concentration used (0.1 mg/mL) falls within the physiologically relevant range for atherosclerotic plaques, where cholesterol content can comprise 10–40% of total plaque mass. The successful detection of cholesterol at this concentration using our modified clinical MRI system demonstrates adequate sensitivity for ex vivo plaque characterization, though quantitative calibration curves relating peak intensity to cholesterol concentration will be necessary for future quantitative analysis.

Our findings align with previous ^1^H-MRS studies demonstrating that spectral signatures of cholesteryl esters are detectable in atherosclerotic tissue [79,80]. However, unlike high-field ex vivo studies that typically employ specialized NMR equipment operating at 7 T or higher field strengths, we demonstrate successful cholesterol detection using a standard 1.5 T clinical MRI system. This represents an important step toward translating spectroscopic plaque characterization from research laboratories to clinical settings. While previous work by Duivenvoorden et al. [80] achieved detection of liquid-phase cholesteryl esters in carotid plaques using ^1^H-MRS, their approach required in vivo imaging with associated challenges of patient motion, blood flow artifacts, and limited spatial resolution. Our ex vivo methodology, though not directly applicable to patient imaging, establishes baseline spectral characteristics that can inform the development of in vivo protocols and provides a controlled environment for validating peak assignments without confounding factors present in living tissue.

### 3.2. MRS in Atherosclerosis Research: Current State and Challenges

MRS and related NMR methods are promising tools for detecting lipid fractions in atherosclerotic plaques, particularly the liquid-phase cholesteryl ester fraction, which correlates with plaque rupture susceptibility [79,80]. In our study, a 1D U-Net model was employed to capture both local peak morphology and global spectral structure. The network effectively suppressed noise, corrected baseline drift, and restored peak symmetry, enabling reconstruction of obscured cholesteryl ester signals [81,82,83,84].

Despite these improvements, current models are trained on HMDB-derived spectra and may not fully capture in vivo complexities such as macromolecular backgrounds, coil-loading effects, J-coupling, or sequence-dependent modulations. Future directions include joint time–frequency domain models, hybrid architectures combining U-Nets with transformers or diffusion models, and integration of uncertainty quantification or self-supervised training to allow direct learning from in vivo data [85,86,87,88,89,90,91].

Clinically, standard 1.5–3 T MRI has limited spectral and spatial resolution, reducing SNR and complicating separation of overlapping lipid resonances. Furthermore, cholesterol crystals, associated with inflammation and plaque instability, require complementary imaging modalities (T1-weighted MRI, μOCT, CT/cryo-imaging) for detection [92,93,94]. Multimodal approaches combining MRS/NMR lipidomics, imaging, and AI analyses improve sensitivity and specificity for plaque composition assessment, and longitudinal studies show that lipid-lowering therapy reduces plaque lipid content detectable via imaging [92,93,94].

Integration of MRS with NMR-based lipidomics enables comprehensive profiling of plaque lipids, identifying metabolite signatures linked to progression and rupture risk, potentially enhancing prognostic value beyond structural imaging alone [78]. High-resolution imaging studies, such as those by Nishimiya et al., highlight cholesterol crystals as a distinct plaque component, underscoring the need for multimodal strategies to distinguish fluid and crystalline phases of cholesteryl esters and capture plaque heterogeneity relevant to clinical risk [95,96,97,98,99].

### 3.3. Study Limitations and Scope

This study demonstrates the feasibility of MRS-based cholesterol detection in atherosclerotic tissue, but several limitations must be noted for clinical translation. First, the ex vivo approach eliminates native tissue architecture and physiological context, and the extended dissolution time (7 days) is impractical for clinical use, highlighting the need for accelerated preparation or in vivo sequences. Second, findings are based on a single plaque specimen; plaque composition varies widely with location, development stage, calcification, and lipid content, so generalizability remains unproven. Third, the study lacks quantitative calibration or validation against gold-standard methods such as GC-MS or histological cholesterol staining, preventing assessment of sensitivity and specificity. These limitations define the path forward: future studies should progressively approximate in vivo conditions using tissue-mimicking phantoms, ex vivo intact vessels, and ultimately in vivo animal and human studies.

### 3.4. Clinical Translation Considerations

Translating these ex vivo findings to clinical practice requires addressing several key challenges. The spectral signatures identified here—particularly peaks at 0.678, 0.876, 1.854, and 5.348 ppm—provide reference standards for developing in vivo sequences. However, distinguishing plaque-derived cholesterol from circulating blood lipids necessitates strategies including cardiac-gated acquisition to minimize pulsatile motion, flow-suppression techniques using pre-saturation pulses, and optimized voxel placement to maximize plaque signal while minimizing blood contamination. Spatial resolution presents another challenge. Our ex vivo 8 × 8 × 8 mm voxel (512 mm^3^) must be balanced against typical in vivo requirements for adequate signal-to-noise ratios. Advanced surface coils designed for vascular imaging—such as carotid phased-array coils—may improve sensitivity and enable smaller voxels. Integration with existing cardiovascular MRI protocols offers a pragmatic path toward clinical adoption, adding 5–10 min to examination time while providing comprehensive plaque characterization when combined with anatomical imaging. Clinical validation would proceed through staged studies: initial comparison of in vivo MRS against ex vivo MRS and histology in carotid endarterectomy patients; prospective natural history studies assessing whether MRS-detected lipid changes predict clinical events; and interventional trials monitoring serial MRS changes with lipid-lowering therapy.

### 3.5. Future Directions: AI-Enhanced Analysis and Multimodal Integration

Emerging AI technologies offer opportunities to enhance MRS-based cholesterol characterization. Deep learning models, including convolutional networks and GANs, can automate peak identification, denoise spectra, and extract quantitative features, enabling regression for cholesterol concentration and classification of plaque stability or rupture risk. Multimodal networks integrating MRS with anatomical MRI or μOCT can capture both metabolic and structural information, while recurrent networks may track temporal plaque changes. Challenges include the need for large labeled datasets, model interpretability, clinical validation, and integration with MRI systems, yet benefits—faster analysis, improved quantification, and enhanced risk stratification—support continued development. While denoising improves SNR and baseline stability, it cannot resolve intrinsically overlapping peaks broadened by plaque heterogeneity or J-coupling. The method is best viewed as a preprocessing step that enhances interpretability but does not substitute for high spectral resolution or advanced acquisition sequences. Future work will focus on training on in vivo plaque spectra to better capture macromolecular backgrounds and coil-loading effects, integrating U-Net with transformers or diffusion models for improved contextual learning, and incorporating uncertainty quantification to flag spectra where denoising may be unreliable.

Nanotechnology-enhanced MRI provides noninvasive imaging of cholesteryl esters and cholesterol crystals, allowing monitoring of plaque changes over time, e.g., in response to statin therapy. Future work should validate methodologies across diverse plaque types, establish quantitative calibration of MRS signals, accelerate extraction protocols, and integrate tissue-mimicking phantoms to bridge ex vivo and in vivo studies. Complementary techniques, including μOCT for crystal visualization, NMR-based lipidomics, and nanoparticle-enhanced MRI, offer multimodal characterization to improve cardiovascular risk assessment and treatment monitoring [79,87,88,89,90,91,92,93,94].

Additionally, 13C MRS enables noninvasive monitoring of lipid composition in tissues and dietary intervention effects, while in vivo ^1^H-MRS provides insight into cardiac metabolism, measuring myocardial lipid content and total creatine levels, which are crucial for energy balance in cardiomyocytes.

Despite its potential, ^1^H-MRS in clinical cardiology is limited by technical challenges, specialized equipment, and expert requirements [95]. Nanotechnology-enhanced MRI offers precise imaging of atherosclerotic plaque lipids, including cholesteryl esters and cholesterol crystals, using targeted nanoparticles. For example, 89Zr-labeled polymeric nanoparticles accumulate in macrophages of ApoE-/- mice plaques, while gadolinium-containing liposomes enhance T1-MRI contrast, enabling monitoring of lipid changes [97,98]. Theranostic nanoparticles, such as HDL-based miNANO, allow simultaneous imaging and removal of cholesterol crystals, stabilizing plaques [99]. Nanoparticles can also detect microcalcifications, indirectly supporting plaque risk assessment [100]. Translational challenges include ensuring specificity to cholesterol, evaluating safety and pharmacokinetics, and achieving sufficient sensitivity for small crystals, potentially requiring high-field magnets or alternative isotopes like 19F-MRI [101,102].

The combination of MRS with advanced imaging techniques such as micro-optical coherence tomography (μOCT) and T1-weighted MRI may create a new, non-invasive strategy for distinguishing between liquid and crystalline phases of cholesterol in atherosclerotic plaques, which is crucial for accurate clinical risk stratification. MRS allows the detection of cholesteryl esters and other lipids in atherosclerotic tissue through characteristic proton signals in the spectra; ex vivo studies have shown that high-resolution proton spectroscopy can identify cholesteryl esters in selected regions of atherosclerotic plaques, and the intensity of lipid signals correlates with their content in the tissue [69,79]. The use of MRS has also been studied in vivo, where the detection of cholesteryl esters in the liquid phase is possible in patients with carotid plaques, confirming the ability of MRS to non-invasively image lipids in human atheroma [70].

High-resolution imaging techniques such as μOCT enable visualization of microstructures of plaques at the subcellular level, including the presence of cholesterol crystals and their relationship with macrophages and other elements of atherosclerotic pathology [103]. Numerous OCT studies have shown that cholesterol crystals are a characteristic feature of unstable plaques and are associated with the presence of macrophages and high-risk features such as lipid-rich necrotic cores and inflammatory cell accumulation [104,105]. Integrating data obtained from μOCT with chemical information from MRS allows for the simultaneous identification of structural crystal formations and their chemical composition, which is not possible with either technique alone.

T1-weighted MRI imaging provides additional information about plaque characteristics by analyzing proton relaxation, which can help differentiate atherosclerotic tissue components based on their relaxation signals. Studies have shown that T1-weighted magnetic resonance imaging techniques are able to identify high-risk plaque features, such as high lipid content and the presence of macrophages, and these findings correlate with OCT features, including the presence of cholesterol crystals and other high-signal-intensity microstructures [106,107]. In addition, the use of T1 and T2* relaxation time mapping in high-field MRI allows for the classification of plaque components—e.g., lipids, hemorrhage, or fibrous tissue—in a more quantitative manner, which is advantageous in assessing their composition [108].

Consequently, the combination of MRS, μOCT, and T1 MRI provides complementary information: MRS provides detailed chemical characterization of cholesterol and its esters, μOCT enables ultrastructural assessment of the presence of crystals and their interaction with inflammatory cells, and T1 MRI provides macroscopic imaging with quantitative mapping of the relaxation properties of individual plaque components. This integrated approach may improve noninvasive clinical risk stratification in atherosclerosis by enabling the identification of plaques rich in crystalline cholesterol—which have been associated with disease progression and increased risk of cardiovascular events—without the need for invasive biopsy or contrast procedures.

## 4. Materials and Methods

### 4.1. Materials

Chloroform was purchased from Sigma-Aldrich (Warsaw, Poland). The study used cholesterol from an official distribution channel and was purchased from Glentham Life Sciences (Corsham, UK). Similarly, chloroform was purchased from Merck’s official distribution channel (Warsaw, Poland). MRS acquisition experiments were performed with a 1.5 Tesla OPTIMA 360 MR manufactured by General Electric Health Care in Munchen, Germany. In this research, we used a balance from Ohaus PX224 (Geifensee, Switzerland). The room temperature during all performed experiments was 18 °C.

### 4.2. Methods

#### Plaque Tissue Sample Preparation for MRS Measurement

Tissue samples of arteries with atherosclerotic lesions were collected by endarterectomies from patients qualified for endarterectomy. Samples were excised from the carotid artery, which had previously been image-diagnosed and finally qualified for removal. All bioethical consents presented in this research were performed according to Bioethical Certificate no 43/2024/B issued by the Bioethics Committee of the Rzeszow Regional Medical Chamber. The collected samples were subjected to in vitro MRS testing. 

The study was conducted using an OPTIMA 360 1.5 Tesla Magnetic Resonance Imaging system manufactured by General Electric Healthcare Technologies, Inc., GEHC (Munchen, Germany). Although this system is excellent for studying the human body, it is not suitable for studying small objects with volumes of 2 mL. Also, coils designed for studying human organs are not suitable in terms of size and shape for Eppendorf tubes. In this study, the receiving system was designed for studying dedicated small objects. The constructed receiving circuit was connected to the MRI system using a factory adapter. Its design was presented in [78] by the same team of researchers. Due to modifications to the receiving system, it was necessary to change the voxel size, which in this case was 8 × 8 × 8 mm (512 mm^3^). This is a significant change compared to spectroscopic sequences in the human body, as the signal is collected from a much smaller volume. Brain spectroscopic studies often involve acquiring signals from a 20 × 20 × 20 mm voxel, which translates to 8000 mm^3^. All measurement was repeated six times. The acquisition of ^1^H-MRS was carried for: (1) 0.2 mg of pure cholesterol was dissolved in 2 mL of chloroform with the use of TR of 1500 ms and TE of 35 ms; and (2) Atherosclerotic plaque tissue immersed in chloroform using TR = 1200 ms and TE = 28 ms. The acquired data from the MRI system were processed in the SAGE7.7.1 software package.

**Figure 7:**
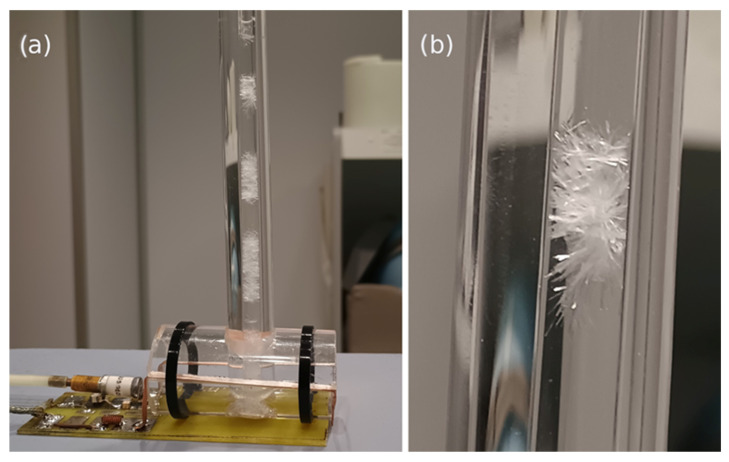
(**a**) The measuring system of test tubes placed in the experimental coil, (**b**) cholesterol crystals.

For the MRS, the standard NMR tube with an outer diameter of 4.95 mm was placed in a slightly larger tube filled with distilled water with a conductivity below 1 μS (Figure 7a). The reason was the need to eliminate errors and artifacts associated with spectroscopic examination at the interfaces of the three media: sample, glass, and air, as well as the need to provide the MRI system with appropriate operating conditions. As these systems are designed for human body examination, their design is tailored to these needs and the properties of the objects being examined. MRI systems, when preparing for scanning, adjust their frequency based on the highest signal. This signal is water, which in the human body is 70% pure. Adding water to the external tube provided the system with the correct signal peak. Figure 7b shows cholesterol crystals formed in the tube as a result of the increased cholesterol concentration.

The seven-day period for sample preparation and cholesterol dissolution in chloroform used in our experiment raises several important issues from both a chemical and a practical clinical application perspective. Prolonged contact between tissue and solvent may lead to partial degradation of sample components. Although cholesterol is relatively stable under organic conditions, other tissue components—such as phospholipids, proteins, and nucleic acids—may undergo hydrolysis, oxidation, or denaturation during multi-day extraction. This can affect the chemical composition of the extract, alter lipid ratios, and lead to the formation of by-products that potentially interfere with MRS spectra or generate additional noise. Consequently, long extraction times can make it difficult to interpret the results in the context of the actual cholesterol content of the tissue in its original state.

From a clinical application perspective, a seven-day sample preparation time is impractical. In diagnostic settings, rapid analysis is required—preferably within a few hours—so that the results can be used in therapeutic decisions. Therefore, prolonged extraction significantly limits the translational potential of the method.

To reduce sample preparation time, several strategies can be considered: mechanical homogenization using Dounce or bead mills (Carl ROTH, Carslrooe, Germany), which increases the contact surface between tissue and solvent and accelerates extraction; intensive mixing or sonication, which improves lipid diffusion; the use of solvent mixtures used in classic Bligh-Dyer or Folch protocols, which enable rapid and effective lipid separation within a few hours; and moderate heating of the sample at a controlled temperature (e.g., 37 °C), which increases the dissolution rate with minimal risk of cholesterol degradation.

Future studies plan to develop accelerated extraction protocols that will reduce sample preparation time to a practical level for clinical applications, limit the degradation of other tissue components, and maintain the repeatability and reliability of cholesterol measurements. This approach will enable standard calibration and possible quantification of cholesterol, which is crucial for further translation of the method into clinical diagnostics.

In summary, a seven-day extraction period is acceptable in ex vivo studies of an evidentiary nature, but is not acceptable in a clinical context. The priority for further research is to implement accelerated extraction methods, mechanical homogenization, and the use of proven chemical protocols that will enable rapid, reliable, and clinically useful determination of cholesterol in atherosclerotic tissues.

The study used cholesterol from an official distribution channel and was purchased from Glentham Life Sciences (Corsham, UK). Similarly, chloroform was purchased from Merck’s official distribution channel.

For compatible spectral standardization and pre-processing, we implemented robust deep-learning (DL)-based denoising of MRS spectra as DL models outperform traditional computational frameworks in various fields, including pre-clinical and medical domains [109,110]. We devised a 1D U-Net deep architecture with three levels of downsampling and symmetric upsampling paths, enabling simultaneous capture of local peak structure and global spectral context [111,112]. Each stage used paired Conv1D–ReLU layers and skip connections to preserve fine-scale metabolic information during denoising. All raw metabolite profiles were standardized using a uniform spectral grid and consistent preprocessing steps. Individual HMDB spectra, originally sampled at variable chemical-shift spacings, were interpolated to a common 1024-point grid spanning the physiologically relevant ^1^H-MRS range of 0–10 ppm. This interpolation ensured uniform dimensionality while preserving peak morphology within the spectral region of interest for lipid and cholesterol analysis. This interpolation ensured uniform dimensionality and preserved peak morphology across spectra. Although most machine-learning approaches operate exclusively in the spectral domain, our denoising pipeline additionally incorporates time-domain modeling of the free induction decay (FID) [113,114,115,116]. Each interpolated spectrum was therefore treated as the real component of a synthetic FID to allow realistic transformations such as apodization, frequency shifts, and complex noise injection. While the U-Net itself operates on frequency-domain spectra, our noise-simulation framework incorporates time-domain transformations of synthetic FIDs to generate realistic training examples. This hybrid approach ensures that the model learns to correct artifacts originating from both domains.

To emulate variations observed across scanners and acquisition protocols, an exponential apodization (line broadening) was applied using

$${FID}_{apod}\left(t\right)=FID(t)e^{-\pi \cdot LB\cdot t}$$

where LB controls the effective line broadening in Hz. The apodized FID was subsequently Fourier transformed using a zero-filled FFT to obtain a spectrum with improved numerical stability for learning. Finally, all spectra—clean and noise-augmented—were normalized to the range [0,1] using global min–max normalization before being reshaped for the 1D U-Net model. This ensured consistent scaling across the training dataset and prevented bias toward high-intensity metabolites.

Because in vivo MRS data are affected by a diverse set of noise sources, a dedicated noise-simulation framework was implemented to generate realistic training examples for the DL denoiser. Individual HMDB peak lists were converted to clean magnitude spectra using a Voigt lineshape model, interpolated to a common 1024-point grid spanning 0–10 ppm, and normalized. These spectra were then used to generate synthetic FIDs for realistic noise augmentation. Each clean HMDB spectrum was augmented with thousands of noisy variants generated from FID-domain perturbations, ensuring that the model learned to correct distortions resembling real, pathological and non-pathological acquisitions. Thermal noise was modeled as complex Gaussian noise added directly to the FID [117]. For a given target signal-to-noise ratio (SNR), noise variance was scaled relative to the clean FID power [118]. Real and imaginary components were sampled independently, reflecting typical receiver characteristics in MRS.

$$\sigma^{2}=\frac{P_{signal}}{2\cdot SNR}$$

**Figure 8:**
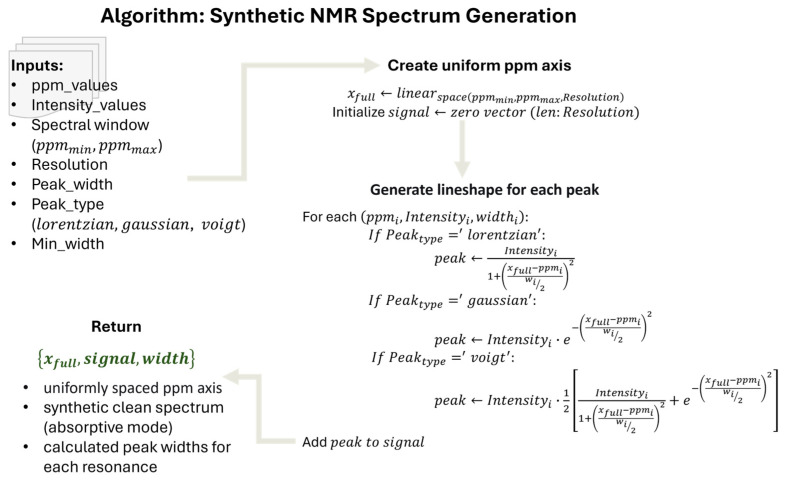
Algorithm for synthetic NMR spectrum generation: Clean spectrum generation from HMDB peak lists using adaptive Voigt lineshapes.

**Figure 9:**
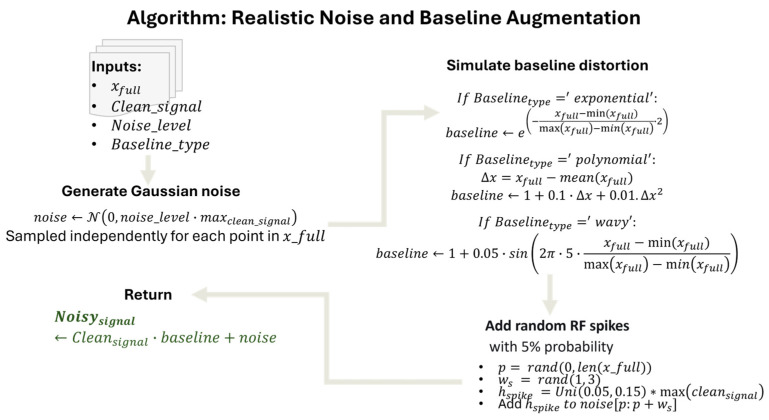
Workflow for generating synthetic NMR spectra with realistic noise and baseline variations: Noise and baseline augmentation via time-domain perturbations with Gaussian noise (thermal noise), Pink (1/f) noise (system-drift). The Polynomial baseline (B_0_ inhomogeneity) added with Random RF spikes (transient interference).

To represent system instabilities and B0-drift-related low-frequency contamination, pink (1/f) noise was synthesized in the frequency domain and inverse-transformed to produce correlated temporal fluctuations [119]. Each synthesized absorptive-mode spectrum was used to generate a synthetic FID via inverse Fourier transform, assuming zero phase for simulation purposes. This synthetic FID was then perturbed in the time domain with realistic noise and apodization functions to create noisy training examples. After perturbation, the FID was Fourier-transformed back to the frequency domain, and the real component (absorptive-mode spectrum) was retained as the noisy input for network training (See Figure 8 for a detailed algorithm of NMR spectral generation from ppm intensity values and Figure 9 for the noise generation algorithm). Independent pink-noise processes were used for real and imaginary channels, allowing the model to learn long-range baseline distortions. Slow-varying background trends were simulated using low-order random polynomials combined with Gaussian-smoothed stochastic drifts, reproducing baseline curvature and undulating backgrounds commonly observed in in vivo spectra [120]. Acquisition-like line broadening was simulated through exponential windowing, producing Lorentzian line-shape changes across the spectrum. These variations helped the model generalize across different scanners and acquisition settings. In total, 10,000 noise-augmented variants per clean HMDB spectrum were generated, creating a large, paired dataset of matched clean/noisy inputs for supervised training of the 1D U-Net denoiser.

Denoising performance was evaluated using six quantitative evaluation metrics, which are defined below. Each metric was computed between denoised and clean (simulated) or between denoised and reference (experimental) spectra.

Signal-to-noise-ratio (SNR): This metric measures the relative strength of the signal compared to the background noise following the equation-$SNR\left(dB\right)=10\cdot \log_{10}\left(\frac{P_{sig}}{P_{nse}}\right)$ where $P_{sig}$ is the mean power of the clean signal, and $P_{nse}$ is the mean power of the noise (estimated as the variance between noisy and clean signals).

Peak SNR (PSNR): It evaluates the fidelity of the denoised signal relative to the maximum possible signal value. It is defined as-$PSNR\left(dB\right)=10\cdot \log_{10}\left(\frac{{MAX}^{2}}{MSE}\right)$ where MAX is the maximum possible intensity value (e.g., 1 after normalization), and MSE is the mean squared error between clean and denoised signals.

Structural similarity index (SSIM): It measures perceptual similarity between two signals, considering luminance, contrast, and structure, following the formula-$SSIM\left(x,y\right)=\frac{\left(2\mu_{x}\mu_{y}+C_{1}\right)\left(2\sigma_{xy}+C_{2}\right)}{\left(\mu_{x}^{2}+\mu_{y}^{2}+C_{1}\right)\left(\sigma_{x}^{2}+\sigma_{y}^{2}+C_{2}\right)}$ where $\mu_{x},\;\mu_{y}$ are means, $\sigma_{x}^{2},\;\sigma_{y}^{2}$ are variances, $\sigma_{xy}$ is covariance, and $C_{1},C_{2}$ are stabilization constants. 0≤SSIM≤1.

Root Mean Square Error (RMSE) quantifies the average magnitude of error between clean and denoised signals. RMSE is defined as-$RMSE=\sqrt{\frac{1}{N}\sum_{i=1}^{N}{\left(x_{i}-y_{i}\right)}^{2}}$.

Mean Absolute Error (MAE) measures the average absolute difference between clean and denoised signals, which follows the formula-$MAE=\frac{1}{N}\sum_{i=1}^{N}\left|x_{i}-y_{i}\right|$.

Pearson Correlation Coefficient (r) evaluates the linear relationship between clean and denoised signals, where $r=\frac{\sum_{i=1}^{N}\left(x_{i}-\bar{x})(y_{i}-\bar{y}\right)}{\sqrt{\sum_{i=1}^{N}{\left(x_{i}-\bar{x}\right)}^{2}}\sqrt{\sum_{i=1}^{N}{\left(y_{i}-\bar{y}\right)}^{2}}}$. −1≤r≤1.

We made a systematic comparison against four classical denoising models as baseline methods, which are described below:

Gaussian smoothing: It implements a Gaussian kernel to smooth the signal, attenuating high-frequency noise. The filtering formula is-$y\left[i\right]=\sum_{j=-k}^{k}x[i+j]\cdot \frac{1}{\sqrt{2\pi \sigma^{2}}}e^{-\frac{j^{2}}{2\sigma^{2}}}$. The standard deviation (σ) is set to 1.5 for our case. It is simple and performs fast noise reduction; however, it blurs sharp peaks.

The Savitzky–Golay filter fits a low-degree polynomial to signal segments via least squares, preserving peak shape better than simple smoothing. For our case, we considered a polynomial order of 3 and 21 points as the window length. The main purpose of using this is to reduce noise while maintaining local spectral features.

Median filtering replaces each point with the median of neighboring points within a sliding window. Our kernel size was fixed at 5. It is known to be robust against impulse noise while preserving edges.

Wavelet denoising decomposes the signal into wavelet coefficients, thresholds the detail coefficients to remove noise, and then reconstructs. The wavelet family that we selected is Daubechies 4 (db4) with decomposition level 3 and soft thresholding. The purpose is multi-resolution noise removal, which is effective for non-stationary noise.

Establishing MRS as a semi-quantitative tool requires correlation with independent reference methods (e.g., enzymatic assay, HPLC) on the same tissue samples. The present study focused on qualitative cholesterol detection. We identify quantitative calibration as an important direction for future validation studies. Translation of ex vivo MRS findings to in vivo clinical applications faces several technical challenges. First, tissue heterogeneity within atherosclerotic plaques—including calcification, fibrous cap, lipid core, and intraplaque hemorrhage—will produce complex, overlapping spectral signatures that may complicate cholesterol-specific peak identification. Second, physiological noise sources, including cardiac pulsation and respiratory motion, will introduce spectral artifacts requiring cardiac and respiratory gating strategies. Third, the proximity of carotid plaques to air-tissue interfaces creates magnetic susceptibility gradients that broaden spectral lines and degrade shimming. Finally, partial volume effects from the relatively large voxel sizes achievable at clinical field strengths may dilute plaque-specific signals with contributions from surrounding blood and vessel wall.

Addressing these challenges will require optimized single-voxel spectroscopy sequences with outer volume suppression, navigator-based motion correction, and potentially higher field strengths (3 T) to improve spectral dispersion and SNR.

## 5. Conclusions

This approach shows potential for assisting in detecting cholesterol-specific spectral signatures in atherosclerotic plaque tissue under ex vivo conditions using magnetic resonance spectroscopy. Through custom-designed receiving coils and optimized acquisition sequences, we successfully identified multiple cholesterol resonances in plaque material that correspond to established NMR reference databases, providing proof-of-concept for MRS-based plaque characterization. The incorporation of a deep-learning denoising framework further enhanced spectral quality by recovering low-intensity cholesterol peaks near the noise floor, thereby improving the visibility and reliability of diagnostically relevant resonances.

MRS and modern imaging methods based on nanotechnology are opening up new perspectives in the diagnosis and risk assessment of atherosclerosis. MRS enables the detection and characterization of cholesteryl esters and the differentiation of the fluid and crystalline phases, which is crucial for assessing plaque susceptibility to rupture. However, the resolution and sensitivity of clinical MRI systems remain limited, requiring further sequence optimization and advanced spectral analysis.

Combining MRS and MRI methods enhanced by nanotechnology allows for increasingly accurate mapping of the lipid and structural composition of atherosclerotic plaques. Integrating these techniques with metabolomic analysis and artificial intelligence may enable the development of precise diagnostic and prognostic biomarkers in the future. However, clinical validation, method standardization, and safety assessment of nanoparticle use in humans remain a key challenge. Only then will these approaches be able to enter routine clinical practice as tools for early diagnosis and monitoring the effectiveness of antiatherosclerotic therapies.

## Figures and Tables

**Table 1:** Stages of cholesterol synthesis.

Stage	Location in the Cell	Substrates/ Products	Main Reactions/ Enzymes	Comments
1. From acetyl coenzyme A to mevalonate	Cytoplasm/ER	Acetyl-CoA → Acetoacetyl-CoA → HMG-CoA → Mevalonate (MVA)	Thiolase (ACAT2): condensation of acetyl-CoA → acetoacetyl-CoA HMG-CoA synthase (HMGCS1): acetoacetyl-CoA → HMG-CoA HMG-CoA reductase (HMGCR): HMG-CoA → mevalonate (rate-limiting step)	Stage sensitive to inhibitors (statins); main regulator of the pathway
2. From mevalonate to isoprenoid units (IPP/DMAPP)	Cytoplasm	Mevalonate → MVA-5-P → MVA-5-PP → IPP ↔ DMAPP	Mevalonate kinase: MVA → MVA-5-P Phosphomevalonate kinase: MVA-5-P → MVA-5-PP Mevalonate-5-pyrophosphate decarboxylase: MVA-5-PP → IPP Isopentenyl pyrophosphate isomerase: IPP ↔ DMAPP	Creation of activated isoprenoid units necessary for further biosynthesis of sterols and other isoprenoid products.
3. From isoprenoid units to squalene	Cytoplasm	DMAPP + IPP → GPP → FPP → Squalene	Prenyl synthases: DMAPP + IPP → GPP → FPP Squalene synthase (FDFT1): 2 × FPP → squalene	FPP is a branching point: sterols vs. non-sterol products (ubiquinone, dolichols, protein prenylation)
4. From squalene to lanosterol	ER	Squalene → 2,3-epoxysqualene → lanosterol	Squalene epoxidase (SQLE): squalene → 2,3-epoxysqualene Lanosterol synthase (LSS): epoxysqualene → lanosterol (steroid cyclization)	SQLE and LSS are potential pharmacological targets; LSS mutations → rare metabolic disorders
5. From lanosterol to cholesterol	ER/ microsomes	Lanosterol → intermediate sterols → Cholesterol	C14 demethylation: CYP51A1 C4 methyl removal: SC4MOL, NSDHL, HSD17B7 Ring modifications: SC5D, EBP, DHCR7, DHCR24, LBR/TM7SF2	Two main pathways: the Bloch pathway (desmosterol → cholesterol) and the Kandutsch-Russell pathway (7-DHC → cholesterol); the final step is catalyzed by DHCR24.
6. Branches/ bypasses	Cytoplasm/ER	24(S),25-epoxycholesterol and other intermediates	Shunt pathway: 24(S),25-epoxycholesterol acts as an LXR ligand	Regulation of cholesterol homeostasis, feedback loops (e.g., HMGCR degradation)

**Table 2:** Comparison of image quality metrics (SNR, PSNR, RMSE, MAE, SSIM, and Pearson’s correlation coefficient) between different traditional denoising methods with 1D U-Net outcomes. Statistical analysis (paired *t*-tests) confirmed that U-Net outperformed all baseline methods (*p* < 0.01).

**Method**	**SNR**		**PSNR**		**RMSE**	
	**Mean**	σ	**Mean**	σ	**Mean**	σ
**Gaussian**	9.0775	2.5109	30.6347	2.2233	0.0303	0.0074
**Savitzky–Golay**	5.9499	1.6453	27.5071	1.4763	0.0427	0.0075
**Wavelet**	13.0381	3.0041	34.5953	2.8215	0.0196	0.0067
**Median**	7.7406	3.0888	29.2978	2.7809	0.0358	0.0101
**U-Net**	**19.1927**	2.8058	**40.7499**	2.8370	**0.0098**	0.0048
**Method**	**MAE**		**SSIM**		**Correlation**	
	**Mean**	σ	**Mean**	σ	**Mean**	σ
**Gaussian**	0.0117	0.0036	0.7692	0.0828	0.9279	0.0393
**Savitzky–Golay**	0.0139	0.0035	0.7651	0.0686	0.8446	0.0646
**Wavelet**	0.0101	0.0035	0.7814	0.0773	0.9741	0.0169
**Median**	0.0129	0.0040	0.7205	0.0932	0.8903	0.0706
**U-Net**	**0.0044**	0.0026	**0.9491**	0.0332	**0.9930**	0.0080

## Data Availability

The original contributions presented in this study are included in the article/Supplementary Materials. Further inquiries can be directed to the corresponding author.

## Supplementary Materials

The following supporting information can be downloaded at: https://www.mdpi.com/article/10.3390/molecules31020352/s1, Table S1: The complete spectral assignment of cholesterol based on high-field (400 MHz) NMR reference data from the Human Metabolome Database (HMDB entry 2491).
