# Supplementary material for: Identification of Cholesterol in Plaques of Atherosclerotic Using Magnetic Resonance Spectroscopy and 1D U-Net Architecture
Source: Molecules. 2026 Jan 19;31(2):352. doi: 10.3390/molecules31020352 (PMC12844486; doi:10.3390/molecules31020352)
Supplement: Supplementary file 1 [file molecules-31-00352-s001.zip › molecules-4046473-supplementary.pdf]

Table S1. Table containing data of Fig. 5 (cholesterol spectrum – 400 MHz NMR

Hz	ppm	intensity	Signal numbers from Fig. 6
2140.26	5.356	88	14
2138.43	5.351	67	14
2137.08	5.348	65	14
2135.01	5.343	81	14
1413.70	3.538	31	13
1412.23	3.534	45	13
1408.08	3.524	54	13
1407.10	3.521	55	13
1402.83	3.511	45	13
1401.25	3.507	35	13
1396.00	3.494	30	13
907.10	2.270	79	12
797.49	1.996	99	11
740.72	1.854	154	10
731.32	1.830	157	9
596.68	1.494	183	8
530.88	1.329	124	7
439.45	1.100	168	6
402.22	1.007	1000	5
368.77	0.923	498	4
362.18	0.907	465	4
349.73	0.876	764	3
347.90	0.871	757	3
343.02	0.859	717	3
341.31	0.855	699	3
276.37	0.692	42	2
270.87	0.678	927	1

.

Table S1 presents the complete spectral assignment of cholesterol based on high-field (400 MHz) NMR reference data from the Human Metabolome Database (HMDB entry 2491). The reference dataset contains fourteen distinct spectral regions corresponding to specific proton environments within the cholesterol molecule.
